# Electrical Stimulation for Immune Modulation in Cancer Treatments

**DOI:** 10.3389/fbioe.2021.795300

**Published:** 2022-01-11

**Authors:** Ritopa Das, Sofia Langou, Thinh T. Le, Pooja Prasad, Feng Lin, Thanh D. Nguyen

**Affiliations:** ^1^ Department of Biomedical Engineering, University of Connecticut, Mansfield, CT, United States; ^2^ Department of Physiology and Neurobiology, University of Connecticut, Mansfield, CT, United States; ^3^ Department of Mechanical Engineering, University of Connecticut, Mansfield, CT, United States; ^4^ Department of Cell and Molecular Biology, University of Connecticut, Mansfield, CT, United States; ^5^ Institute of Materials Science, University of Connecticut, Mansfield, CT, United States

**Keywords:** immunotherapy, electrical stimulation, electroporation, cancer treatment, immunostimulant, immunogenic cell death

## Abstract

Immunotherapy is becoming a very common treatment for cancer, using approaches like checkpoint inhibition, T cell transfer therapy, monoclonal antibodies and cancer vaccination. However, these approaches involve high doses of immune therapeutics with problematic side effects. A promising approach to reducing the dose of immunotherapeutic agents given to a cancer patient is to combine it with electrical stimulation, which can act in two ways; it can either modulate the immune system to produce the immune cytokines and agents in the patient’s body or it can increase the cellular uptake of these immune agents via electroporation. Electrical stimulation in form of direct current has been shown to reduce tumor sizes in immune-competent mice while having no effect on tumor sizes in immune-deficient mice. Several studies have used nano-pulsed electrical stimulations to activate the immune system and drive it against tumor cells. This approach has been utilized for different types of cancers, like fibrosarcoma, hepatocellular carcinoma, human papillomavirus etc. Another common approach is to combine electrochemotherapy with immune modulation, either by inducing immunogenic cell death or injecting immunostimulants that increase the effectiveness of the treatments. Several therapies utilize electroporation to deliver immunostimulants (like genes encoded with cytokine producing sequences, cancer specific antigens or fragments of anti-tumor toxins) more effectively. Lastly, electrical stimulation of the vagus nerve can trigger production and activation of anti-tumor immune cells and immune reactions. Hence, the use of electrical stimulation to modulate the immune system in different ways can be a promising approach to treat cancer.

## Introduction

### Overall View

Immunotherapy is emerging as a very promising approach for cancer treatment. So far, numerous approaches of immunotherapy have been developed. Some of these are to use checkpoint inhibition, T cell transfer therapy, monoclonal antibodies and cancer vaccination. Very often, immunotherapy is utilized as a part of a combinatory therapy along with other treatments like radiation, chemotherapy, remission surgery etc.

Despite the successes of different immunotherapy approaches, most of them involve high doses of chemical or biological reagents which pose significant concerns on problematic side effects and toxicity. Therefore, the use of physical cues or electrical signals to modulate and stimulate immune response could offer a safer alternative for immune modulation and engineering. Electrical stimulation could modulate the immune system to produce the needed endogenous cytokines, consequently eliminating the need to dose the patient with these immune agents externally. Also, approaches like electroporation make the delivery of immune agents more effectively, hence reducing the dose of these drugs. Yet, there is still little-to-no attention on this new and exciting field of using electrical stimulation (ES) for immune therapy. Here, in this review, we will first briefly present the current state of art immune therapy for cancer treatment, using traditional biochemical cues, and then go into detailed descriptions on the science and application of different ES approaches on the field of immune therapy.

### Role of Immunotherapy in Cancer Treatment Thus far

Immunotherapy is a type of treatment of any disease or ailment by activating or suppressing certain parts of the immune system. In some cases, the immune system can be controlled or modulated to attack certain target cells. This process is referred to as immune engineering. Immune Engineering and immunotherapy have been used for multiple purposes like tissue regeneration, wound healing, vaccination, cancer treatment, allergy treatment etc. (Calvet and Mir, 2016; Elias et al., 2017; James and Bernstein, 2017; Hua and Bergers, 2019; Oliveira et al., 2019)

Immunotherapy is becoming increasingly popular for treatment of cancer. In most cancer patients, the immune systems are not able to recognize and target cancer cells. This is because cancer cells start as normal healthy cells that change or alter and start to proliferate uncontrollably. Since cancer cells actually develop from normal cells, the immune system doesn’t always recognize them as foreign. However, the immune system can be manipulated in a controlled fashion to attack and destroy cancer cells.

For instance, a common approach is to use immune checkpoint inhibitors. Immune checkpoints are normally present in the immune system and keep immune responses from being too strong or killing off native cells of the body. By blocking these checkpoints, these drugs allow immune cells to respond more strongly to the cancer cells (C. Azoury et al., 2015; Wu et al., 2017; Abdel-Wahab et al., 2018). The work of F. Stephen Hodi et al. (Hodi et al., 2010) showed that blocking the CTLA-4 (cytotoxic T-lymphocyte–associated antigen 4) Immune Checkpoint Pathway can be used in the treatment of metastatic melanoma. In this work, the authors used the antibodies Ipilimumab to activate the CTLA-4 antigen in patients with metastatic melanoma and showed that the use of the drug increased the survival period of the patients. Another commonly targeted checkpoint inhibitor is TIM3 (T-cell immunoglobulin and mucin domain-3) which enhances T cell production and activity (Friedlaender et al., 2019). Similarly, the work of Chrystelle Brignone et al. (Brignone et al., 2010) shows the combination of paclitaxel and Eftilagimod alpha to block *the* LAG-3 (lymphocyte agitation gene-3) Checkpoint Pathway helps in the treatment of metastatic breast carcinoma and increases the survival of patients. Many other similar antigens and drugs have been used for *modulating the* PD-1 Immune Checkpoint Pathway and *T-Cell-Activating Pathways* (Beatty et al., 2011). Several drugs targeting both PD-1 and CTLA-4 are being used extensively for clinical trials (De Miguel and Calvo, 2020) including Nivolumab, Pembrolizumab, Atezolizumab and ipilimumab. Several other works have reported using novel checkpoints such as NKG2A which activates natural killer cells and T cells. This therapy has also been combined with therapeutic antibodies to attack metastases (Creelan and Antonia, 2019). Clinical trials have also been conducted using MK420 against LAG-3 checkpoint (De Miguel and Calvo, 2020). Monoclonal antibodies can be designed in the lab to target and bind to specific cancer cells. Once injected into the body, these antibodies attach to the cancer cells and mark them as targets for the immune system (Scott et al., 2012; Pento, 2017; Parakh et al., 2020). In 1997, rituximab developed by IDEC pharmaceuticals became the first approved monoclonal antibody for the treatment of low-grade B cell lymphoma (Leget and Czuczman, 1998; Scott, 1998; Grillo-López et al., 2002). Since then, around 30 monoclonal antibodies (Table 1) have received approval from the United States Food and Drug Administration (US FDA) for the treatment of a variety of solid tumors and hematological malignancies (Boyiadzis and Foon, 2008; Scott et al., 2012; Lu et al., 2020).

**TABLE 1:** List of existing FDA-approved monoclonal antibodies for cancer treatment. (Reichert, 2012; Ecker et al., 2015; Reichert, 2016; Reichert, 2017; Kaplon and Reichert, 2018; Kaplon and Reichert, 2019; Kaplon et al., 2020; Kaplon and Reichert, 2021).

Commercial name	Scientific name	Cancer type	Format	FDA approval
Zynlonta	Loncastuximab tesirine	Large B-cell lymphoma	Humanized IgG1 ADC	2021
Jemperli	Dostarlimab	Endometrial cancer	Humanized IgG4	2021
MARGENZA	Margetuximab	HER2+ breast cancer	Chimeric IgG1	2020
BLENREP	Belantamab mafodotin	Multiple myeloma	Humanized IgG1 ADC	2020
Monjuvi	Tafasitamab	Large B-cell lymphoma	Humanized IgG1	2020
Sarclisa	Isatuximab	Multiple myeloma	Chimeric IgG1	2020
Enhertu	[fam]-trastuzumabderuxtecan	HER2+ breast cancer	Humanized IgG1 ADC	2019
Padcev	Enfortumab vedotin	Urothelial cancer	Human IgG1 ADC	2019
Polivy	Polatuzumab vedotin	Large B-cell lymphoma	Humanized IgG1 ADC	2019
Lumoxiti	Moxetumomab pasudotox	Hairy cell leukemia	Murine IgG1 dsFv immunotoxin	2018
Poteligeo	Mogamulizumab	Cutaneous T-cell lymphoma	Humanized IgG1	2018
IMFINZI	Durvalumab	Bladder cancer	Human IgG1	2017
Bavencio	Avelumab	Merkel cell carcinoma	Human IgG1	2017
Tecentriq	Atezolizumab	Bladder cancer	Humanized IgG1	2016
Lartruvo	Olaratumab	Soft tissue sarcoma	Human IgG1	2016
Portrazza	Necitumumab	Non-small cell lung cancer	Human IgG1	2015
Opdivo	Nivolumab	Melanoma, non-small cell lung cancer	Human IgG4	2014
Blincyto	Blinatumomab	Acute lymphoblastic leukemia	Murine bispecific tandem scFv	2014
Cyramza	Ramucirumab	Gastric cancer	Human IgG1	2014
Kadcyla	Ado-trastuzumab emtansine	Breast cancer	Humanized IgG1, ADC	2013
Gazyva	Obinutuzumab	Chronic lymphocytic leukemia	Human IgG1	2013
Perjeta	Pertuzumab	Breast cancer	Humanized IgG1	2012
Yervoy	Ipilimumab	Metastatic melanoma	Human IgG1	2011
Arzerra	Ofatumumab	Chronic lymphocytic leukemia	Human IgG1	2009
Vectibix	Panitumumab	Colorectal cancer	Human IgG1	2006
Avastin	Bevacizumab	Colorectal cancer	Humanized IgG1	2004
Erbitux	Cetuximab	Colorectal cancer	Chimeric IgG1	2004
Bexxar	Tositumomab-I131	Non-Hodgkin lymphoma	Murine IgG2a	2003
Zevalin	Ibritumomab tiuxetan	Non-Hodgkin lymphoma	Murine IgG1	2002
Herceptin	Trastuzumab	Breast cancer	Humanized IgG1	1998
Rituxan	Rituximab	Non-Hodgkin lymphoma	Intravenous	1997

Another immune-modulating approach is T-cell transfer therapy. In this treatment, immune cells are collected from the tumor tissues. These cells are sorted for the most active ones against cancer cells. These highly activated cells are selected, expanded and put back into the patient through a needle in a vein. This treatment boosts the natural ability of T cells to fight cancer. For instance, in the works of Yee et al. (2002), T cells targeting the tumor-associated antigens, MART1/MelanA and gp100 were used to treat patients with refractory, metastatic melanoma, and the results showed that use of these T cells were safe and gave clinically favorable outcomes. Engineering T cells to express suicide molecules and tumor antigen specific receptors is also a common therapeutic practice. Bonini et al. (1997) published an article in 1997 in which they treated patients with lymphoma by bone marrow transplant using native donor bone marrow and bone marrow containing lymphocytes transduced with the herpes simplex virus thymidine kinase (HSV-TK) suicide gene. Their results showed that most of the patients receiving native donor bone marrow suffered from relapse of their cancer but the engineered lymphocytes helped to provide positive antitumor outcomes in most of the patients receiving that treatment. Also, Park et al. (Schönmann et al., 1986; Park et al., 2007) developed a tumor specific, single chain antibody-derived, chimeric antigen receptor designated CE7R for re-directing the antigen-specific effector functioning of cytolytic T lymphocytes. These cells were infused in children with recurrent/refractory neuroblastoma. Their results showed a robust anti-tumor cytotoxic activity of the infused T cells. Recently, Kang et al. (2020) developed nanoparticles that mimic T cells and can bypass the immunosuppressing environment in a tumor.

Oncolytic virotherapy is another effective form of immunotherapy that has been used to treat cancer in the past. The earliest records of oncolytic activity date back to 1912 when De Pace discovered an improved condition in a cervical cancer patient who had received Pasteur’s rabies vaccine after a dog bite (De Pace, 1912). Today their functions are still being explored in terms of immunotherapeutic effect for cancer treatment. Oncolytic viruses (OV) are unique in that they have the ability to replicate within cancer cells, ultimately leading to the cells’ destruction (Alzahrani et al., 2019). In addition, they induce an immunogenic type of cell death and exert immune system modulation. The viruses can be genetically engineered for specific tumor cells or can be naturally occurring (Lin and Nemunaitis, 2004; Lundstrom, 2018). There are various types of oncolytic viruses from different virus families including Herpesviridae, Adenoviridae, Poxviridae, Paramyxoviridae, etc. So far, oncolytic virotherapy has been tested on treating a number of different cancer modalities like melanomas, prostrate cancer, myelomas, lung cancers etc. (Macedo et al., 2020).

Lastly, treatment vaccines and immune system modulators are also used widely. Both of these enhance the body’s immune response to cancer by modulating specific parts of the immune system or the system as a whole. Cancer vaccines come in form of different modalities and agents. A common type is tumor cell vaccines that are derived from cancerous cells. The tumor cells in the vaccine can be autologous i.e., derived from the patient themselves (Schulof et al., 1988; Harris et al., 2000; Berger et al., 2007) or allogenic i.e., derived from secondary human tumor cell lines (Sondak et al., 2006; Kelly and Giaccone, 2011; Wang et al., 2011; Van Den Eertwegh et al., 2012). Another common cell-based cancer vaccine is to use dendritic cells, which are often generated or modified *ex vivo* to be injected into cancer patients (Inaba et al., 1992; Rosenblatt et al., 2011; Guo et al., 2012). Non-cell-based options include protein/peptide-based vaccines that are also used for cancer treatment (Buonaguro et al., 2011; Schwartzentruber et al., 2011; Schiffman and Wacholder, 2012). Lastly, genetic materials like DNA or RNA plasmids are also used as cancer vaccines (Weide et al., 2009; Orlandi et al., 2011; Aurisicchio and Ciliberto, 2012). Immune modulators, similar to vaccines, activate the immune system to attack tumor cells. One of the primary types of immune modulators are cytokine like Interferons (Li et al., 2009; Isorce et al., 2015; Kistner et al., 2017) and Interleukins (Fry et al., 2001; Melchionda et al., 2005; Zhang et al., 2005). Apart from that, BCG or *Bacillus* Calmette-Guérin is used broadly for treatment of gut cancer and a few other types of cancers (Murahata and Mitchell, 1976; Pettenati and Ingersoll, 2018). Lastly, immunomodulatory drugs that modify biological responses are also used for cancer therapies (Piccolomo et al., 2020; Wang et al., 2021).

### The Use of Electrical Stimulation for Medicine and Immune Modulation

#### The Use of ES in Medicine

Electrical stimulations have, for a long time, been used for many different medical applications like fracture healing, nervous stimulation, muscular stimulation, etc. This is because electrical fields of different natures and magnitudes can react with the cells/ions in the body fluids and other biochemical factors to modulate different physiological processes (Hieda and Nam, 2013; Lin, 2016). Physiological electric fields (EFs) are important factors that control and adjust the cellular and tissue homeostasis. Our body generates a biological EF ranging between 10 and 60 mV at various different locations (Foulds and Barker, 1983). These electrical fields are very important in wound healing, tissue regeneration and several other physiological processes. When any tissue is wounded, a steady direct current (DC) EF is initiated around the damaged tissue. This endogenous EF guides migration of cells toward the edge of the wound, ultimately leading to healing due to cell migration and facilitating repair into the wounded tissue (Song et al., 2002). The same mechanism can be applicable for the regeneration of other damaged tissues like bones, cartilages, ligaments, tendons, skin etc. Hence, over the years, the use of external electrical stimulation to improve and enhance regeneration of tissues and healing of wounds has become a common practice. For instance, in 1953, Yasuda *et al.* published a work where they applied continuous electrical current to a rabbit femur for 3 weeks and demonstrated new bone formation around the cathode (Ryaby, 1998). Different types of electrical stimulators, both external and implantable, have been developed. For external stimulators, studies have been performed by placing electrodes externally around the affected area to provide stimulation (Pettine et al., 1993; Dmochowski et al., 2019; Knutson et al., 2019). For implantable stimulators, scaffolds have been fabricated using a number of polarizable materials with inherent or induced surface charge (Zealear et al., 2003; Charthad et al., 2018; Das et al., 2020). Also, the effects of electrical fields of different natures, like continuous (direct current) (Mobini et al., 2016; Jang et al., 2018; Molsberger and Mccaig, 2018) or pulsed electrical fields (Feedar et al., 1991; Zizic et al., 1995; Thompson et al., 2010; Das et al., 2020) have been studied extensively. Each type of electrical stimulation plays a distinctly different role in our body. Most importantly, the use of different magnitudes of electrical fields have been established to achieve different biomedical purposes. For instance, use of low voltage electric fields (10–100 mV) can be used for cell proliferation, migration and tissue regeneration applications (Hou et al., 2011; Love et al., 2018; Szewczyk et al., 2019; Das et al., 2020). On the other hand, higher voltage electrical fields (above 1 kV) can be used to initiate electroporation, apoptosis, etc. which can be used to target and destroy diseased tissue in conditions like cancer (Davis et al., 1986; Miller et al., 1988; Love et al., 2018). Hence, by varying the magnitude and the nature of application, electrical stimulation can be used in many different medical applications.

#### The Use of Electrical Stimulation for Immune Modulation

Just like any other cells in the body, immune cells are also affected by the application of ES. ES can enhance immune-cell proliferation, secretion of cytokines, extracellular matrix production, and vascular development (Ferrigno et al., 2020). Presence of continuous as well as pulsed electrical fields can affect macrophages, B cells and T cells. For instance, electrical field modulation has been shown to affect the polarization of macrophages into (Itoh et al., 2006; Nakamura et al., 2007; Huang et al., 2018) M1 or M2 subtypes (Hoare et al., 2016; Li et al., 2016; Oliveira et al., 2019). K. M. C. Oliveira et al. (Oliveira et al., 2019) published a work in 2019 in which they studied the effects of electrical stimulation on macrophage polarization in a rat amputated limb model. In this work, they provided ES into the amputated stump using an implanted stimulation device. Their results show that animals that had received ES had higher numbers of both M1 and M2 polarized macrophages in their limbs. Also, as a result of this they observed formation of new blood vessels in animals that received the ES whereas the control and sham animals didn’t show new vessel formation. Compared to macrophages, less studies were focused on the effects of electrical fields and electrical stimulations on B cells and T cells. One work by Arnold et al. (2019) showed how exogenous electrical fields affected the migration, proliferation and cytokine production of T cells. All the other studies usually talk about the effects of physiological electric fields on B cells and T cells, like electrical activities resulting from Mitogens (Hu et al., 1990) or nerves (Straub et al., 2008; Rosas-Ballina et al., 2011) or the epithelium tissue (Arnold et al., 2019).

Interestingly, ES was also supposed to improve respiratory function in COVID-19 patients, inhibit SARS-CoV-2 growth, boost immunity, reduce pain, and improve the penetration of antiviral drugs (Allawadhi et al., 2020). Especially, ES has been used widely in the form of electroporation, wherein it can increase drug uptake by increasing the permeability of the cell membrane. Electrical stimulation combined with DNA vaccination has been discovered to have various effects on immune modulation. A study conducted by Bachy et al. demonstrated that electrical pulses increased the immunogenicity of an influenza DNA vaccine which was injected intramuscularly in BALB/c mice (Bachy et al., 2001). However, the most common application of electrical stimulation to modulate immune system is still for cancer treatment. In the following sections, we will provide detailed review on this aspect.

## Electrical Stimulation to Modulate the Immune System for Cancer Treatment

### Electrical Stimulation Using Direct Current

Electrotherapy by low level direct current (current flowing in one direction only) has been found to have antitumor effects in various murine models (Humphrey and Seal, 1959; David et al., 1985; Marino et al., 1986; Heiberg et al., 1991; Samuelsson et al., 1991; Griffin et al., 1994) and in clinical trials (Plesnicar et al., 1994; Xin, 1994). Most of the tumors can be reduced or eradicated with direct current of long duration, and the appropriate spacing of multiple electrodes in the tumor (Sersa and Miklavcic, 1993). The spacing of the electrodes near the tumor is critical and determines the extent of tumor retardation. In a paper published by Miklavcic et al. (1997) similar findings were found. In this work, LPB (lipopolysaccharide binding protein) tumors were inoculated subcutaneously in syngeneic CS7BV6 mice and immunodeficient Swiss nude mice (nu/nu). The electrotherapy was conducted when the tumor reached 80 mm^3^ in C57BV6 mice and 40 mm^3^ in nu/nu mice. A single shot of electrotherapy by 0.6 or 1.0 mA current for a 1-h duration was performed on each animal. In addition to electrotherapy, genetically engineered Chinese hamster ovary (CHO) cells were selected for the secretion of high levels of interleukin-2 (lL-2) and intratumorally or peritumorally injected into both types of mice. Electrotherapy by both 0.6 and 1.0 mA in immunocompetent mice yielded significant tumor growth delay as compared to the immunodeficient mice, demonstrating that the immune system is an important component that aids in the effectiveness of electrotherapy. For the animals that received the CHO cells injection, combining electrotherapy with immunotherapy resulted in significantly higher number of cures and more tumor disappearances as compared to immunotherapy alone. This confirms the superior effect of combining direct current stimulation and IL-2 injection on the tumor treatment, compared to the use of each therapy alone.

### Nano Pulse Electrical Stimulation

**FIGURE 1:**
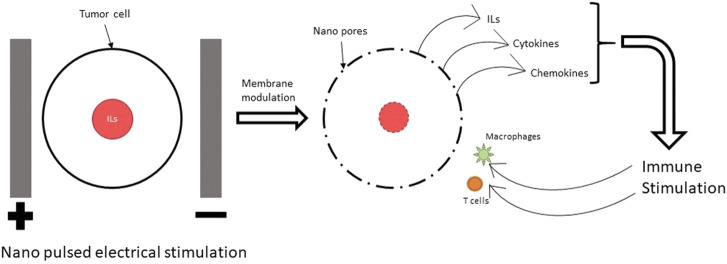
Mechanism of immune modulation using NPS. The nano pulses of electric field lead to electroporation (creation of pores in the cell and nuclear membrane) in cancer cells. This leads to release of ILs, cytokines and chemokines which trigger the immune system to attack the cancer cells.

Nano pulse electrical stimulation (ES) refers to a type of stimulation using electrical pulses which are only a few nano seconds long. This is the most common ES used in combination with immunotherapy for cancer therapy (Ren et al., 2013; Miao et al., 2015; Nuccitelli et al., 2017; Skeate et al., 2018; Zhang et al., 2019). This pulsed ES is effective against cancers by synergistic effect of electroporation and activation of the immune system. The electrical stimulation of a high frequency attacks the cancer cells and creates pores in their cell membranes. As a result, the cell organelle and parts of the cell nucleus, like the DNA spills out of the cells. These cell organelle and DNA stimulate the immune cells in the vicinity and activate them to attack the cancer cells in the tumors as shown in Figure 1.

There has been extensive works to use nano pulse stimulation (NPS) for treatment of cancer. Nuccitelli et al. published an article in 2017 in which they studied the effects of NPS at different voltages (12–30 kV/cm) on three different cancer cell lines (MCA205 or mouse fibrosarcoma cells, McA-RH7777 or rat hepatocellular carcinoma and Jurkat E6-1or human leukemia cells) (Nuccitelli et al., 2017). Their results show that initiation of cell death or apoptosis in the cultured cells is greatest at 15 kV/cm and requires 50 A/cm^2^ indicating this magnitude of the electric field to be most effective in triggering self-destruction of the tumor. They measured the level of Caspase-3 activation (which is a crucial mediator of programmed cell death) 3 h after the NPS treatment and observed that activated caspase-3 increases 8-fold in Jurkat E6-1 cells and 40% in the MCA205 and McA-RH7777 cells. They also looked for markers of immunogenic cell death (ICD) like ecto-calreticulin (CRT), ATP and HMGB1 24 h port treatment. Expressions of all three markers increased with the use of NPS treatment as compared to controls and was comparable to samples treated with anthracycline (a common chemotherapy drug). The authors hypothesize that NPS is a Type II inducer of ICD since it causes stress in the mitochondria and endoplasmic reticulum. Additionally, if CRT is produced early in the cell death process, it leads to an ‘eat-me’ response, initiating phagocytosis.

Zhang et al. reported their work in 2019 the use of NPS (30 kV/cm, 100 ns, 200p) on a mouse malignant melanoma model (Zhang et al., 2019). Their results showed that the numbers of T lymphocytes as measured in the spleen were increased, indicating that the NPS had stimulated the immune system of the animal to attack the tumor. CD3^+^ CD4^+^ T cells and CD3^+^CD8+T cells (which are both killer T cells) were shown to increase whereas regulatory T cells and myeloid-derived suppressor cells (which regulate and suppress other immune cells) were shown to decrease. In addition, the levels of TNF-α and IL-2 (which are tumor antigens that help attract immune cells to the site of the tumor) were increased and the level of IL-10 (which facilitates cancer metastasis) was decreased.

Lastly, Chen et al. reported the use of NPS on two different tumor models-murine hepatocellular carcinoma (HCC) and canine osteosarcoma (Chen et al., 2017). For the canine osteosarcoma model, their results show that tumor volume and serum alkaline phosphatase in animals that received NPS were lower than the controls and almost comparable to animals that were treated with amputation (standard treatment for this type of cancer). Survival percentage in the animals receiving NPS was much higher than controls and comparable to animals receiving amputations. Also, the number of cases with metastasis and the rate of metastasis reduced with the NPS treatment. Similarly, for the murine model with transplanted hepatocellular carcinoma with high metastatic potential, their results show a much lower tumor volume for animals receiving NPS as compared to the controls. The number of cases with metastasis and the rate of metastasis were reduced with the NPS treatment.

### Electrochemotherapy

#### Overall Review on the Electrochemotherapy

**FIGURE 2:**
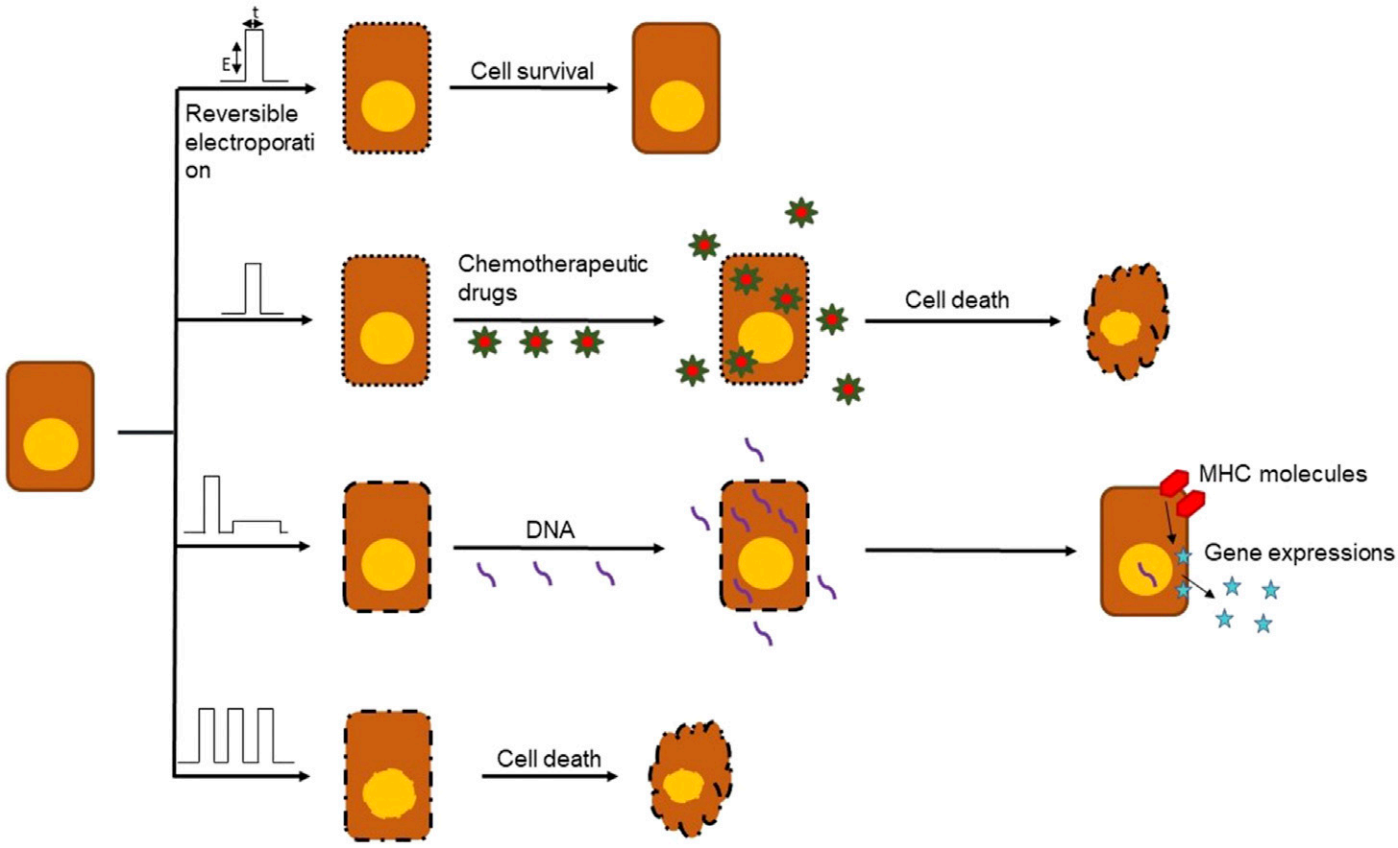
Different outcomes of electroporation based on type, frequency, amplitude and number of EPs. Electroporation can be used in a number of ways to damage cancerous cells like delivery of chemotherapy drugs, DNA vaccines and cell death.

Recent studies have shown that there are benefits of using electrochemotherapy (ECT) in combination with immunotherapy for cancer treatment. Anti-tumor ECT uses electroporation specifically to accomplish this. Electroporation is a term used to describe the phenomenon of increased permeability of the cell membrane after the application of short and intense electric pulses, known as EPs. It uses electricity to manipulate the cells and target tissues, and is an effective and safe technique that is currently being used to transfer materials such as nucleic acids, cytotoxic drugs, and ions into target cancerous cells and tissues (Escoffre et al., 2009; Breton and Mir, 2012) as shown in Figure 2.

Anti-tumor electrochemotherapy (ECT) is also a way to increase anti-cancer drug uptake by means of electroporation. ECT is non-ablative and non-thermal local treatment of solid tumors consisting in the application of EPs combined with administration of non/low-permeant anti-cancer molecules (Mir, 2006; Breton and Mir, 2012; Escoffre and Rols, 2012). The EPs are delivered locally to the whole volume of the nodule, and reversibly permeabilize the cells without killing them. Then the anti-cancer drug is administered into the tumor and can enter the target cells and achieve its cytotoxic activity without restrictions. ECT is advantageous because it selectively targets tumor cells by applying EPs locally and also using an anti-cancer drug that displays specific cytotoxicity towards the dividing cancer cells. ECT can be used to target immune cells as well to induce immune modulation (we will go into more details of that in the subsequent sections). However, for ECT to be efficient, the following conditions are required; sufficient concentration of drug has to be present in the tumor, and the whole tumor must be covered by a permeabilizing electric field. When these conditions are present, ECT eliminates the cancer cells while sparing normal cells and histological structures (Calvet and Mir, 2016).

In 2006 the multicentric European Standard Operating Procedures of ECT (ESOPE) study was conducted, and it established the standard operating procedures for ECT to be used in the clinic (Marty et al., 2006; Gehl et al., 2018). The study reported that a train of eight EPs of 100 μs and of appropriate field amplitude, between 3 and 10 kV, had to be delivered using either invasive or non-invasive electrodes, depending on the size and depth of the nodules to treat. In addition, the study reported that complete tumor regression was seen in 73.7% of the treated nodules, and the overall objective response was 84.8% 6 months after one ECT session. Finally, the study emphasized the efficiency and safety of the procedure, specifically when bleomycin was injected intravenously. There have been many other clinical trials performed, and overall the rate of complete tumor regressions after one single ECT treatment is 60% (Miklavčič et al., 2014). There were also rarely local relapses (suggesting that once a certain region of the body is treated with ECT, there are very narrow chances of the cancer returning to the same place as the original cancer), which indicates the local long-lasting response to ECT. In recent years, electrodes in form of microneedle arrays have been developed for a easier application of ECT on patients (Esmaeili and Friebe, 2019). As of 2016, pulsed electromagnetic fields have also been used for ECT in a mouse melanoma mode demonstrating successful cancer treatment (Kranjc et al., 2016; Esmaeili and Friebe, 2019). There were minimal side effects of ECT which included edema, erythema, superficial epidermal erosion, relative pain and muscle contraction (Miklavčič et al., 2014).

#### ECT Induced Immunogenic Cell Death

ECT strategies are often focused on immune stimulation, and interestingly there has been evidence that the immune system also contributed to ECT efficiency. ECT mediated tumor regression decreased dramatically in animals exempt of functional T lymphocytes when compared to immunocompetent mice (Mir et al., 1991; Mir et al., 1992; Sersˇa et al., 1997). Also, the edema following EP delivery on tumors was less severe in immunodeficient mice than in immunocompetent one, which further suggests that EP-mediated effects depend on the presence of an intact immune system. This edema plays an important role as it increases vascular permeability, most likely allowing for local infiltrates of Dendritic cells (DCs) (Roux et al., 2008; Gerlini et al., 2013) and lymphocytes (Mekid et al., 2003). In a study done by Gerlini et al. (2013), the anti-tumor immunity effects of ECT were addressed by investigating the presence of dendritic cells (DCs) in the inflammatory infiltration of ECT-treated lesions. ECT was administered on melanoma patients by injecting them with Bleomycin followed by cutaneous stimulation with EPs of variable amplitude with 1–5,000 Hz delivery frequencies. Biopsies from patients (n = 9) were taken before ECT (T0), at day 7 and day 14 after treatment and they were studied by immunofluorescence for DCs-related antibodies. Before treatment, Epidermal Langerin^+^ cells (LCs) were the most represented subset of immune cells. At day 7, ECT induced a significant reduction in epidermal LCs number while at day 14 they were completely replaced. It was noted that the few LCs seen to be intermingled with metastatic melanoma cells at T0 decreased after treatment, suggesting that ECT induced the activation of LCs. Similarly, at day 1 after ECT, LCs were found to express CCR7 in three patients, which mediate LCs differentiation to CD83, the typical DCs maturation marker, and to regional lymph nodes.

**FIGURE 3:**
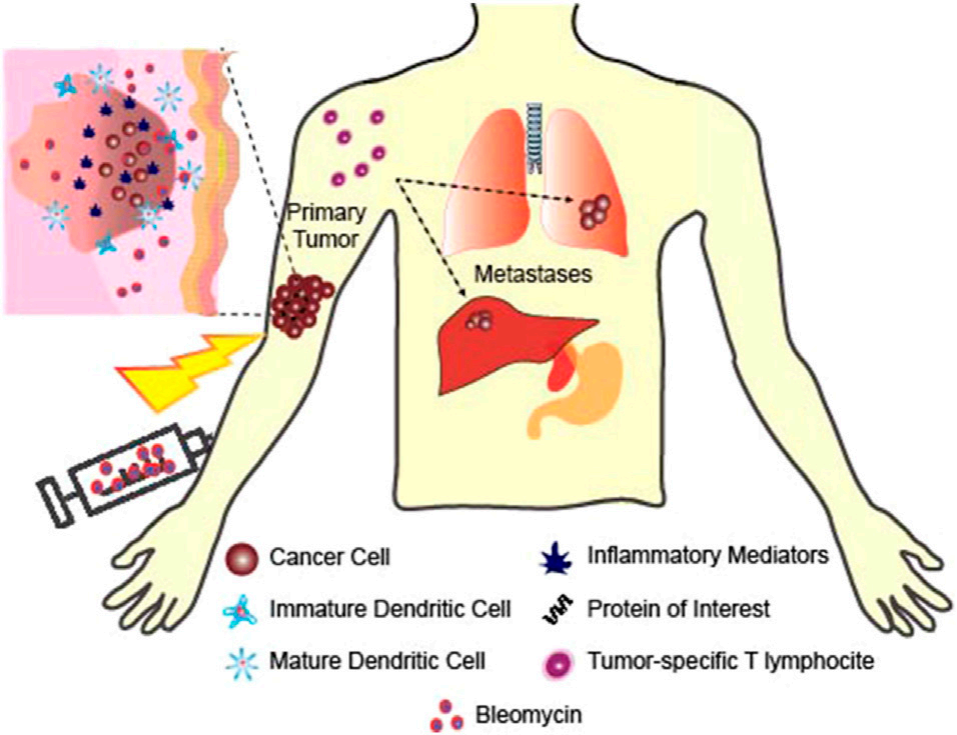
Healing of non-targeted and contralateral tumor using ECT. ECT creates tumor specific immune cells that have the ability to kill cancer cells including the non-ECT sensitive tumor cells and tumor cells far from the ECT treated area.

Sersa et al. demonstrated that an anti-tumor activity of circulating monocytes and splenic T lymphocytes was elicited in mice after ECT treatment of murine SA-1 fibrosarcoma (Sersa et al., 1996). The study showed immune system activation after treatment, and that electrical pulses increase the effectiveness of the chemotherapeutic drugs by permeabilizing tumor cells and creating “holes” or openings for the drugs to enter. The drug used for these experiments was bleomycin (BLM). 5 mg/kg (100 µg per mouse) of bleomycin was injected intravenously into the A/J mice and electrical pulses (8 square wave pulses of 1.04 kV amplitude, 100 us pulse width and 1 Hz frequency) were additionally delivered to some of the mice by two flat and parallel stainless-steel electrodes placed 8 mm apart at the opposite margins of the fibrosarcoma SA-1 tumor. Treatment with BLM or electrical pulses alone induced moderate antitumor effect. However, treatment was significantly more effective with combined BLM and ECT. It was found that 52% of the tumors responded completely in 120 days, and were determined cured. The remaining 10 tumors regrew with some delay after approximately 10 days in partial response. Increased immune resistance was evaluated by determining the phagocytic activity and the ability to elicit oxidative bursts by monocytes and polymorphonuclear granulocytes after ECT. The percentage of monocytes that were able to elicit oxidative burst was increased 7 days after the ECT treatment but then returned to normal after 14 days. Immune responses were measured by blast transformation tests of spleen mononuclear cells after electrochemotherapy, and it was found that T lymphocyte activity increased 14 days after ECT. White blood cell count and the number of monocytes were also increased. These generated T cells could potentially have the ability to kill non-ECT-sensitive cancer cells within the primary tumor, limit metastatic spreading, and be responsible for the absence of local relapses (Kroemer et al., 2013) as shown in Figure 3.

Recently, Tremble et al. published a work using ECT with Cisplatin in murine models of lung and colorectal cancer (Tremble et al., 2019). Their results show that ECT with Cisplatin treatment enabled a much lower growth rate of cells CT26, B16F10, LLC and Pan02 both *in vitro* and *in vivo* as compared to electrical stimulation alone or cisplatin alone. Immune cell infiltration into the tumor was also studied by the authors in the CT26 tumors. They found high numbers of CD11c^+^ DC cells, F4/80^+^ macrophages, NIMP-R14^+^ neutrophils, CD19^+^ B cells and DX5^+^/CD3^-^ natural killer cells. Their results also showed significantly lower metastasis of the tumors to the lungs from the ECT with Cisplatin treatment as compared to the treatments with electrical stimulation alone or cisplatin alone. This is a clear indication that ECT with Cisplatin was able to activate immune cell production in the spleen and kills tumors both at the primary and secondary (metastasis) sites.

#### ECT in Combination With Immune-Stimulating Agents

ECT is unique in that it combines electroporation with a low-dose chemotherapy which results in enhanced cytotoxicity. ECT is used for local treatment for metastases of a variety of cancers, and it has been found that in combination with immunostimulating agents, ECT leads to a systemic tumor response (Goggins and Khachemoune, 2019). Preclinical evidence suggests that the association of ECT along with immunostimulating agents could be an efficient way to cure the targeted malignant tumors and any distant nontargeted tumor nodules, even if it is an undetectable metastasis (Figure 3).

A study by Mir et al. (1995) used a non-permanent cytotoxic drug of bleomycin (BLM) and cell permeabilizing electric pulses (8 pulses of 1.35 kV/cm and 100 us at a frequency of 1 Hz), with IL2-based immunotherapy to treat murine LPB sarcoma tumors. IL2 is a T cell proliferation factor and a cytokine that has anti-tumor properties. The mice used in this study had two tumors, one near the site of treatment to serve as the primary tumor and a second one far from the site of treatment to serve as the contralateral tumor. ECT was combined with an intratumoral administration of histocompatible IL2-secreting cells. The IL-2 secreting cells were first injected directly into the peritumoral edema evident roughly 24 h after ECT. Electrical pulses were delivered 3 min after the BLM injection and from the day after ECT, equivalent number of cells releasing around 2,000 U of IL-2 were injected zero, one, two, or three times a day for 5 days. Three daily injections for 5 days increased the number of cures from about 40–60% to 80–90% suggesting that local injections of tiny amounts of IL-2 secreting cells could help improve ECT effectiveness. Repeated cell injections were necessary due to their short *in vivo* half-life. Mice that received the injections in addition to ECT demonstrated a significantly higher rate of complete regression and lower tumor volume than mice that received ECT alone. In addition, it generated a systemic response as anti-tumor effects were seen in contralateral non-ECT treated tumors. With the combination treatment, contralateral non-ECT-treated tumors were seen to be highly infiltrated by CD4^+^ and CD8^+^ T lymphocytes, which were believed to be responsible for the observed 50% tumor rejection rate of these untreated contralateral tumors (Figure 3).

Aside from the IL2-based immunotherapies, TLR9 ligands, such as CpG oligodeoxynucleotides (CpG ODN), have also been tested in combination with ECT. CpG oligodeoxynucleotides (or CpG ODN) are short single-stranded synthetic DNA molecules that contain a cytosine triphosphate deoxynucleotide (“C”) followed by a guanine triphosphate deoxynucleotide (“G”). They are known to induce Th1 immune response. Roux et al. (2008) published a work in 2007 where they combined CpG ODN injections with ECT to treat LPB and B16OVA melanoma tumors. For the ECT, they used Bleomycin with eight square electric pulses of 100 us and 1,300 V/cm delivered at a frequency of 5,000 Hz. Their results show that the combination of CpG ODN with ECT increased the number of TLR cells in both the tumor models. The tumor volumes were also seen to reduce drastically in both tumor models under the CpG ODN/ECT treatments as compared to ECT alone or CpG ODN injection alone. Hence, this clearly concludes that injection of the CpG ODN into ECT-treated tumors dramatically increased the treatment efficiency in immunocompetent mice.

In the past few years, there have been studies regarding the efficacy of ECT with immunotherapy as treatment of metastatic melanoma (Goggins and Khachemoune, 2019). A case report published by Brizio et al., in 2015 discovered that ECT in combination with ipilimumab, a CTLA-4 inhibitor, resulted in complete cutaneous and visceral response of the 28 tumor nodules treated (Brizio et al., 2015). Similarly, a retrospective analysis conducted by Mozzillo et al. found a local response in 67% of patients and a systemic response in 60% (Mozzillo et al., 2015). A 2016 study compared ECT in combination with ipilimumab and Nivolumab (a PD-1 inhibitor) and an overall conclusion that ECT plus PD-1 inhibitors were more effective than ECT plus CTLA-4 inhibitors (Heppt et al., 2016). Finally, a 2017 case report conducted by Karaca et al. found complete cutaneous and visceral response when using ECT in combination with Nivolumab to treat metastatic melanoma (Karaca et al., 2018).

### Electro-Gene Transfer Aided Cancer Treatment

Gene therapy for cancer consists of administration of a DNA/RNA encoding an antigen of interest in order to protect the body against pathogens or cancer cells exposing this antigen, and has been developed for a wide range of applications (Kutzler and Weiner, 2008). The encoded antigen will eventually generate a pool of specific B and T cells, some of which will remain as memory cells serving in long-term protection. Tumor-specific CD8^+^ T cell generation is desired in the context of anti-cancer therapy due to their association with the secretion of TH1 cytokines (e.g., tumor necrosis factor *a* (TNFα) and interferon γ (IFNγ)) (Vesely et al., 2011; Braumüller et al., 2013). The level of expression of the antigen by injecting the encoded plasmid is one of the key factors influencing the outcome and success of this technique. Despite the success of results obtained in small rodents with the administration of naked plasmids, poor gene uptake by target cells was one of the primary reasons why there was a failure in translating these promising results to humans (Liu and Ulmer, 2005; Kutzler and Weiner, 2008). However, studies done in rodent models and larger animals have shown that the efficiency of gene transfers was greatly increased by EP when compared to the plasmid injections alone. One example of this refers to a DNA encoding the prostate-specific antigen (PSA) in the context of prostate cancer. Roos et al. revealed that antigen expression was enhanced by up to 1000-fold when DNA injections was combined with EP, which also led to an improved PSA-specific T cell priming (Roos et al., 2006).

A paper published by Goto et al. (Goto et al., 2000) demonstrated the use of Electro-gene transfer (EGT) for treating murine solid tumors. Plasmids containing genes such as the “A” fragment of the diphtheria toxic (DT-A) or herpes simplex virus thymidine kinase (HS-tk) were transferred into tumors in mice using *in vivo* EP. The mice were male, BALByc mice and murine tumors were injected into the flank of the mice via an inoculum of 0.1 ml of serum-free culture medium containing 1 × 10^6^ cells. For EGT, a needle array electrode was used to deliver eight square-wave pulses at a frequency of 1 Hz, with a pulse length of 50 ms and 33 V. The first type of therapeutic gene tested with EGT was an expression plasmid for DT-A that generates toxicity through its inhibition of protein synthesis, leading to apoptotic death of a tumor cell expressing the gene (Palmiter et al., 1987; Yagi et al., 1990). The average tumor volumes in control group were larger than those in the treated group at day 16 and its suppression rate was 30%. However, this efficacy isn’t enough for treatment of malignant tumors *in vivo*. The other therapeutic gene tested was an expression plasmid for the HS-tk gene. It was determined that the percent suppression of tumor growth within the treatment group in all of the experiments was more than those with DT-A treatment. In both groups it was more efficient when the gene was present along with EGT. Hence, HS-tk was proved to be more effective as a DNA vaccine in combination with EGT than DT-A.

In a study conducted by Roos et al. (2006), the induction of PSA-specific CD8^+^ T cells in mice to a prostate cancer DNA vaccination encoding prostate-specific antigen (PSA) after intradermal electroporation was evaluated. Various electroporation conditions were compared based on their ability to induce PSA-specific CD8^+^ T cell responses. Large number of CD8^+^ T cells were produced in the spleens of mice under different electroporation conditions. The levels of PSA-specific CD8^+^ T cells were higher in the C57Bl/6 mice immunized under the following conditions: 750 V/cm, six pulses, 100 As (condition A), bimodal condition E and the low pulses alone (condition D), and combination of 1125 V/cm, two pulses, 50As + 275 V/cm, 8 pulses, 10 ms (condition E). It was determined that DNA vaccination in electroporation conditions resulted in a significant increase in the levels of PSA-specific T cells, when compared to DNA deliver done without electroporation.

Besides DNA, Messenger RNAs (mRNAs) have been shown to be efficient tools in cancer immune-gene therapy. Electroporation has also been commonly used to deliver mRNAs into targeted cells (Van Tendeloo et al., 2001; Van Bockstaele et al., 2008; Hashimoto and Takemoto, 2015; Gerer et al., 2017). A recent study conducted by Van Hoecke et al. (2018) used a mRNA encoded for a mixed lineage kinase domain-like (MLKL) protein which is a necroptosis executioner. The authors used the MLKL-mRNA against melanoma and colon carcinoma models, and employed electroporation for the intratumoral delivery of the mRNA. Their results showed increased tumor cell death as well as reduced tumor size and increased instances of survival for animals receiving MLKL-mRNA. Additionally, the delivery of the MLKL-mRNA enhanced antitumor immunity against neo-epitopes by activating CD8α^+^ dendritic cells and type I interferons (IFN). These results indicate that electroporation led to an efficient uptake of the MLKL-mRNA by the tumor cells and the effectiveness of the antitumor effects of the MLKL protein.

#### Electroporation Based Cytokine Therapy

**FIGURE 4:**
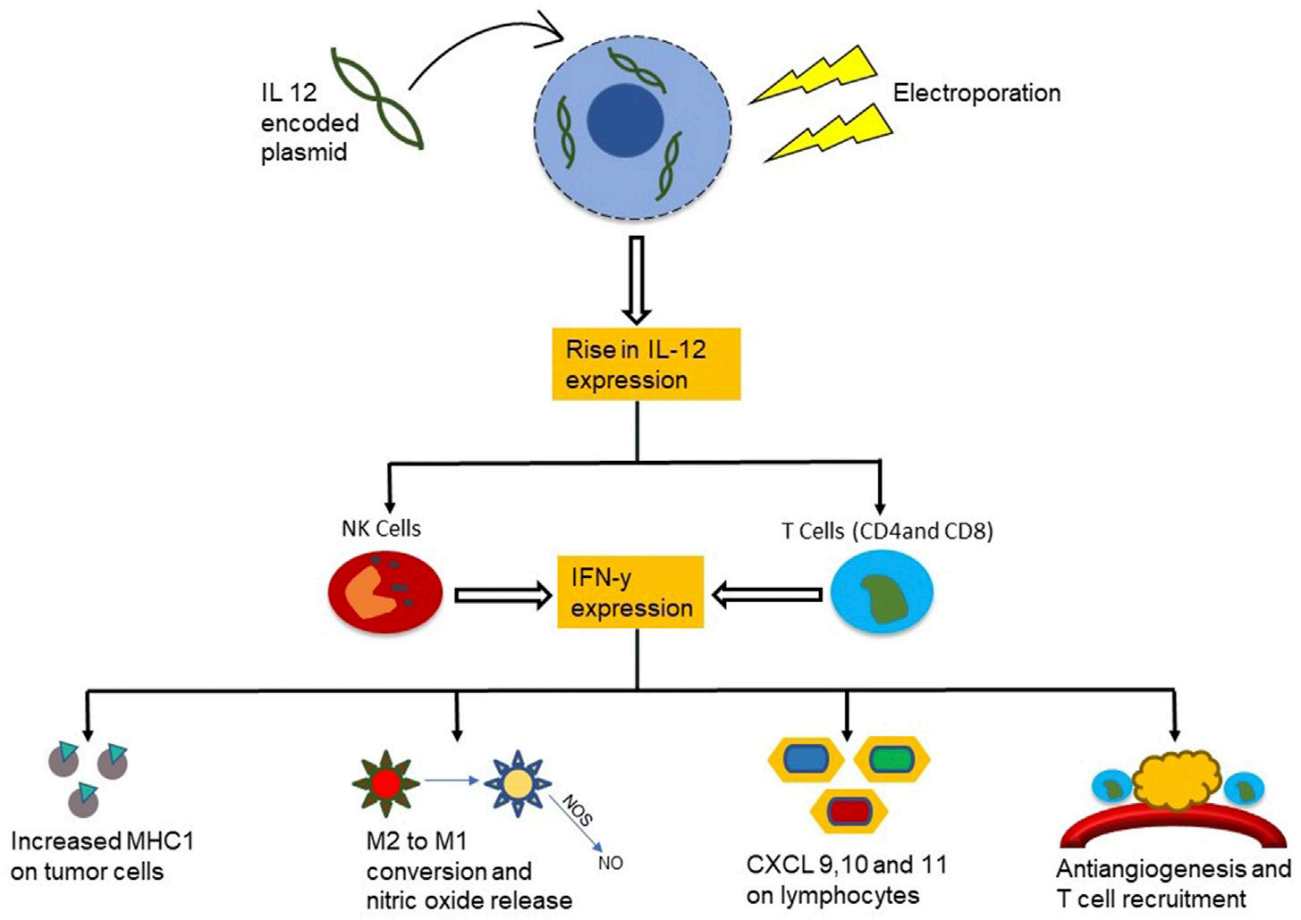
Mechanism of the functioning of cytokine IL-12 in combination with electroporation in cancer treatment. The IL-12 plasmid uptake into the cancer cell is higher after electroporation which upregulates the IL-12 release near the tumor. This attracts NK cells and T cells to the cancerous region leading to increased IFN-y expression and a cascade of anti-cancer immune activity.

Cytokine therapy in combination with electroporation has proven to be a successful and safe technique to treat cancer (Chopra and Satkauskas, 2018). Specifically, therapy that utilizes electroporation and cytokines such as IL-12 can enhance the effectiveness of immunotherapy (Kishida et al., 2001; Lohr et al., 2001; Yamashita et al., 2001; Heller et al., 2006; Cemazar et al., 2010) due to easier cytokine plasmid uptake and increased fragility of the cancer cells. Interleukin-12 (IL-12) is a cytokine produced by phagocytic cells, B cells and dendritic cells which play a crucial role in the interaction between the adaptive and innate arms of immunity (Trinchieri, 1995). IL-12 has been a very promising candidate for fighting against cancer due to its ability to induce production of other cytokines such as IFN-γ (Colombo and Trinchieri, 2002). In addition, it stimulates CD4^+^ T cell differentiation into TH1 cells and stimulates cytotoxic functions of NK cells, NKT cells, and CD8^+^ T cells. IFN-γ production also in turn encourages the production of IL-12 by phagocytes and dendritic cells (Ma et al., 1996), hence acting as a positive feedback mechanism and leading to a powerful defense response against intracellular pathogens. As electroporation increases IL-12 plasmid uptake and IL-12 production, this mechanism becomes more powerful (Figure 4).

An example for the application of ES-enabled cytokine therapy is to treat mast cell tumors (MCT) which are the most common malignant cutaneous tumors in dogs and account for 21% of cutaneous tumors. Current treatment for MCT depends on whether or not the MCT is well-differentiated, and has a low success rate. The goal of a study produced by Pavlin et al. was to evaluate the local antitumor effect, the systemic transgene release and side effects of Electrogene therapy (EGT) performed with therapeutic plasmids encoding human IL-12 in canine MCT (Pavlin et al., 2011). A plasmid encoding human IL-12 was injected into nodules of MCT in 8 patients, and was followed by application of electrical pulses. Electric pulses (EPs) were applied using needle electrode arrays (2 arrays each composed of 4 electrodes with a 4-mm distance between them). The EP delivery style was one high voltage pulse (1 × 1200 V/cm, 100 µs), immediately followed by 8 low voltage pulses (8 × 50 ms, 140 V/cm, 2 Hz). 11 tumor nodules were treated with EGT in 8 patients and after 1 month of EGT most tumors had decreased in size and the tumors were either in a stable state, or completely responsive to treatment. There was also an increase in leukocytes in the IL-12 and EGT treated tumors, which were not present in the untreated tumors. The appearance of side effects was monitored with weekly clinical examinations, which consisted of bloodwork. A complete blood count and a biochemistry panel were conducted, and the results were all within normal parameters suggesting that there were no adverse side effects of the treatment. Cytokine concentrations were increased, specifically IL-12 (∼12 pg/ml over 28 days) and IFN gamma (∼165 pg/ml over 28 days), after treatment indicating that immune cells had become more active. Hence this study shows that not only is the combination of IL12 with electroporation an effective way of treating MCTs, it is a very viable and safe treatment with minimal side effects.

In a study conducted by Daud et al. (2008), plasmid IL-12 electroporation was carried out in patients with metastatic melanoma. They received electroporation on days 1, 5, and 8 during a single 39-day cycle, into metastatic melanoma lesions with a total of six 100-µs pulses at a 1,300-V/cm electric field through a six-electrode array following DNA injection. Pre- and post-treatment biopsies were acquired for histological evaluation to determine the IL-12 protein levels. A total of 24 patients were treated at seven dose levels, and showed minimum systemic toxicity. Post-treatment biopsies revealed an increase in IL-12 protein levels (proportional to the dose of plasmid injections given), and higher tumor necrosis and lymphocytic infiltration. 52% of patients (8 patients) showed disease stabilization or partial response after treatment, supporting that IL-12 in combination with electroporation is safe, effective, reproducible and titratable.

Even though IL12 is one of the most commonly used immune cytokine for electroporation based cancer therapies, other cytokines that have also been used are other interleukins like IL 18, IL 33 and IL 15 as well as interferons like IFn-α and IFn-γ (Chopra and Satkauskas, 2018). Each of these cytokines has been used for electroporation based cytokine therapy by activating production of T cells, Th1 cells differentiation, increasing antigen presentation and recruitment of dendritic cells.

### Electrical Stimulation of Vagus Nerve (VNS) for Immune Modulation

The vagus nerve, otherwise known as cranial nerve X, is a major component of the autonomic nervous system (Howland, 2014). It plays a crucial role in the regulation of metabolic homeostasis by controlling heart rate, gastrointestinal motility and secretion, pancreatic endocrine and exocrine secretion, hepatic glucose production, and other visceral functions. In addition, it is a major constituent of the inflammatory reflex, which controls innate immune responses and inflammation during pathogen invasion and tissue injury (Pavlov and Tracey, 2012).

Cranial nerve X is formed via a series of nerve rootlets from the lateral portions of the medulla oblongata. It exits the cranium through the jugular foramen. It has a superior ganglion, a jugular ganglion, an inferior ganglion, and the nodose ganglion which allows for visceral and special sensation (Cuoco et al., 2016). The vagus nerve is able to respond to multiple environmental stimuli via receptors such as those for mechanical stretching, pressure, osmotic pressure, temperature change, and pain.

Electrical stimulation of the vagus nerve has shown to be successful in studies performed on the cardiac tissues, circulatory system, nervous system, and digestive system. The vagus nerve, sometimes called the wandering nerve, senses peripheral inflammation, and generates action potentials through the vagal efferents, resulting in an inhibition of the increasing levels of proinflammatory cytokines (Meregnani et al., 2011; Sun et al., 2013; Payne et al., 2019).

Various studies have proposed that through its anti-inflammatory properties, vagus nerve stimulation in modulation of the immune system slows down tumorigenesis, thus suggesting it could potentially contribute to cancer treatments in the future.

**FIGURE 5:**
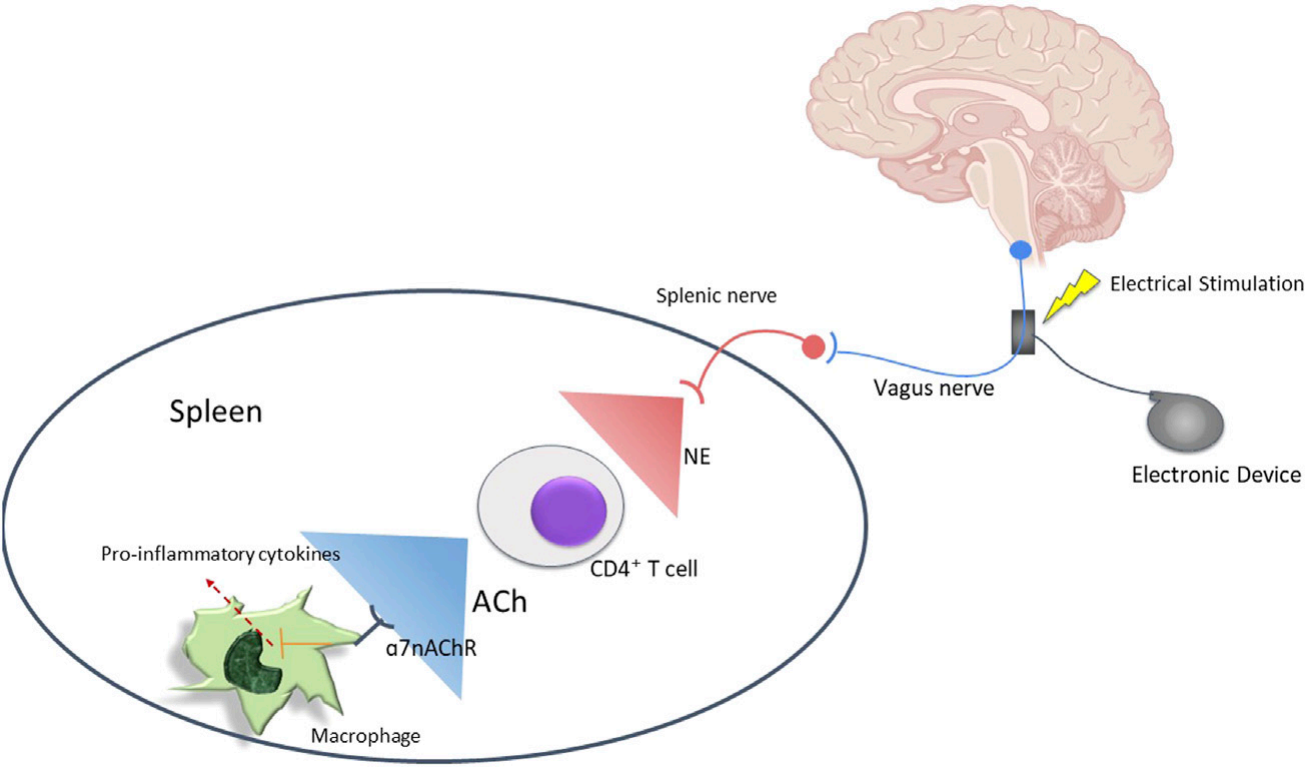
Mechanism of immunomodulation by the Vagus nerve. Vagus nerve can be stimulated to induce T cells to release acetylcholine which can increase macrophage production and activity.

Inflammatory responses require regulations to prevent excessive inflammation. Ideally, such regulations or anti-inflammatory responses are rapid, reversible, localized, and adaptable to changes in inflammatory input signals (Jarczyk et al., 2019). The nervous system’s ability to rapidly communicate and respond makes it ideal to mediate these anti-inflammatory responses. By sensing cytokines and other inflammatory products, afferent vagal neurons are able to convey inflammatory status in tissues to the NTS (nucleus tractus solitarius). This results in the activation of efferent vagal neurons through the vagovagal reflex. Electrical stimulation of vagal neurons in rodents inhibited the systemic increase in levels of proinflammatory cytokines, and this pathway was termed as the cholinergic anti-inflammatory pathway (Suzuki and Nakai, 2018). Upon activation, efferent vagus nerve fibers suppress cytokine production via the release of acetylcholine (ACh) from CD4^+^ T cells. Acetylcholine interacts with the alpha-7 nicotinic acetylcholine receptor macrophages which initiate an anti-inflammatory response (Jarczyk et al., 2019) as shown in Figure 5.

The tumor microenvironment (TME) contains innate immune cells (macrophages, myeloid-derived suppressor cells, dendritic cells, and natural killer cells) and adaptive immune cells such as T- and B- lymphocytes (De Visser and Coussens, 2006). Tumor-infiltrating immune cells can inhibit or support tumor growth. Tumor-infiltrating lymphocytes control tumor growth, however tumors often subvert their activity and support the differentiation of tumor-associated macrophages which promote tumor growth (Whiteside, 1999; Radoja and Frey, 2000; Qian and Pollard, 2010). Tumor-associated macrophages (TAM) are present at all stages of tumor progression. M1 (“classically” activated macrophages) are potent killers of pathogens and tumor cells, while M2 (“alternatively” activated macrophages) are typically associated with a pro-tumoral role (Mantovani et al., 2004). Due to the fact that most of the immune cells express the α7nAChR it was predicted and proven that VNS affects anticancer immunity (since it is well established that VNS is highly responsible for AChR production). A study performed by *Guarini et al.* indicated that activation of parasympathetic nervous system by VNS served as a suppressor mechanism of NF-κB activation, a signaling pathway that plays an important role in cancer-related inflammation (Greten et al., 2004; Pikarsky et al., 2004). Normally, immature myeloid cells generated in bone marrow differentiate into mature macrophages, granulocytes, or dendritic cells. In pathological conditions such as cancer however, differentiation of IMCs into mature myeloid cells is partially blocked. Expansion of the IMC population typically results in the suppression of T-responses in the TME, due to the upregulation of immune suppressive factors, and the increased production of nitric oxide and reactive oxygen species. Myeloid progenitors that give rise to these cells, known as MDSCs, have been shown to have reduced proliferation and expansion upon overexpression of Trefoil Factor 2 (TFF_2_) (Gabrilovich and Nagaraj, 2009). Additionally, overexpression of TTF2 has shown to remarkably suppress tumor growth (Dubeykovskaya et al., 2012).

Natural killer (NK) cells are another avenue through which VNS can be used for cancer treatment. These cells become functionally impaired by inhibitory factors such as TGF-β1 in TME (Ghiringhelli et al., 2005; Viel et al., 2016). However, research done by *Wang et al.* suggests that stimulation of the transcutaneous vagus nerve reduced levels of TGF-β1 in ventricular tissue and peripheral plasma (Wang et al., 2015) which increases the activity of NK cells. NK cells can then mediate antitumor responses through various mechanisms such as establishing direct cytotoxic interactions with target cells, that could result in killing numerous cancer cells, or inducing tumor elimination though receptor-mediated pathways (Johnsen et al., 1999; Vermijlen et al., 2002; Deguine et al., 2010). Activated NK cells produce pro-inflammatory cytokines and chemokines that can promote innate and adaptive immune responses and direct antitumor activity. They are also immunoregulatory cells that modulate the activity of T-cells and dendritic cells and allow them to further affect tumorigenesis.

Lastly, various studies have reported how vagal signaling alters the function of T-lymphocytes, and research suggests that the vagus nerve exerts a down-regulatory effect on CD4^+^ T-cell function. This suppresses the activity of Treg cells which repress T cell differentiation to cytotoxic T-lymphocytes (CTLs), resulting in lower numbers of CTLs and limiting immunity against tumor cells (Fukaura et al., 1996; Roncarolo et al., 2001). Dendritic cells are known as potent antigen presenting cells (APCs) which can uptake, process, and present tumor antigens to naive T-cells (Shurin, 1996; Banchereau and Steinman, 1998; Steinman and Banchereau, 2007). Dendritic cells (DCs) along with vagus nerve stimulation help differentiate naive CD4^+^ and CD8^+^ T-cells into antigen-specific T-cells. CD4^+^ T-cells become T helper cells which assist in the differentiation of B-cells into antibody secreting cells which downregulate the functions of CTLs. DCs are crucial for inducing and maintaining antitumor immunity but in the tumor environment their antigen-presenting function may be lost or inefficient. Additionally, tumors can interfere with DC maturation (Steinbrink et al., 1997; Fiorentino et al., 2016). Hence, the upregulation of T cell differentiation due to VNS helps combat with all these factors and produce more CTLs to attack the tumor and kill cancer cells.

## Conclusion

In brief, we have described the use of electrical stimulation for immune modulation and engineering for the treatment of cancer. Based on the nature and different modes of electrical stimulation, the mechanism of immune modulation and tumor suppression vary greatly. For instance, steady direct current is used (albeit very rarely) for activating the immune system in general so that tumor cells are recognized and attacked. Pulsed electrical stimulation is used for a variety of applications, the most common of which is electroporation (EP). Specifically, nano second long electrical pulses of high intensity were used for a more permanent pore formation in cells, leading to the cancer cells death. This effect also acts as a beacon for immune cells like T cells, macrophages, natural killer cells, dendritic cells and other phagocytotic cells that get recruited to the cancerous sites and attack the remaining tumor. On the other hand, treatments like Electrochemotherapy (ECT) and gene vaccination use EPs of longer duration and lower intensities for a transient electroporation which is then used as a way to deliver treatment agents like chemotherapeutic drugs, anti-cancerous DNA fragments, cytokine-encoded mRNA and cytokine encoded plasmids. Very often, a lot of these techniques are combined with additional immunostimulants to make the immunogenic effect more robust. Lastly, electrical stimulation of vagus nerves can help to suppress inflammation and maneuver certain parts of the immune system to attack cancerous cells.

Electrical stimulation has definitely brought in a new and more sophisticated angle for cancer treatment. Traditional treatments of cancer with the most successful outcomes include use of chemotherapeutic drugs or immunotherapeutic agents like cytokines, immunomodulating agents, genetic vaccines or T cell-based therapies. These treatments, while being effective, require injection of very high doses of chemical and biological agents into a patient leading to a number of negative side effects. Electrical stimulations, especially those using electroporation serve the dual purpose of activating the immune system and increasing the permeability of cells to the therapeutics and immune agents-hence reducing the requirement of high doses of toxic injections needed for cancer therapy (Denet et al., 2004; Escobar-Chávez et al., 2009).

However, electrical stimulation, especially at the high voltages necessary for electroporation, has certain drawbacks. For instance, it can activate the apoptotic phase in healthy cells (Rubinsky, 2007). Also, high voltage electrical stimulations will cause involuntary and often unnecessary muscle spasms which can cause fatigue, wear and tear in muscles as well as a lot of discomfort in patients (Denet et al., 2004). Moreover, site specificity and the efficacy of delivering therapeutic and immune agents are still challenges with electrical stimulation (Tsoneva et al., 2010). So far, most of the electrical stimulation techniques to modulate or activate the immune system have only been tested in animals with a very few clinical trials so far. In addition, most of the studies involve external or subcutaneous electrical stimulators. These types of electrical stimulators have lower efficiency since the electric pulses need to travel through thick tissue layers (e.g., skin, muscle, bone etc.) to reach the cancerous region. Only a few studies have used implantable electrical stimulators. Yet, most of these implantable stimulators are non-biodegradable and contain batteries that can release toxic chemicals into the body. Furthermore, a revision surgery is needed to remove the stimulator from the body-which cause a lot of trauma for a patient to endure. Hence there is a need to develop effective biocompatible and biodegradable electrical stimulators that can degrade and cause no harm on the body over time, thus eliminating the requirement for a revision surgery. These types of implantable and biodegradable electrical stimulators are a very promising direction for future studies in immunotherapy for cancer treatment since they increase the efficiency of the treatments and also eliminate the need of revision surgeries. As more knowledge are obtained in the field of immunology and more advanced electrical stimulators are developed, immune engineering using electrical stimulation may become an increasingly popular approach for cancer therapy.
